# Both Manuka and Non-Manuka Honey Types Inhibit Antibiotic Resistant Wound-Infecting Bacteria

**DOI:** 10.3390/antibiotics11081132

**Published:** 2022-08-20

**Authors:** Samantha R. Hewett, Stephany D. Crabtrey, Esther E. Dodson, C. Alexander Rieth, Richard M. Tarkka, Kari Naylor

**Affiliations:** 1 Department of Biology, University of Central Arkansas, Conway, AR 72035, USA; 2 Department of Chemistry, University of Central Arkansas, Conway, AR 72035, USA

**Keywords:** antibacterial, alternative treatments, honey, antibiotic resistance, minimum inhibitory concentration

## Abstract

Postoperative infections are a major concern in United States hospitals, accounting for roughly 20% of all hospital-acquired infections yearly. Wound-infecting bacteria, in particular, have a high rate of drug resistance (up to 65%), creating life-threatening complications. Manuka honey, native to New Zealand, has been FDA-approved for wound treatment in the United States after studies demonstrated its ability to inhibit a variety of bacterial species and facilitate wound healing. The aim of this study was to identify alternative (non-manuka) honey types that can be specifically used against antibiotic resistance bacteria in wound infections. We utilized a honey-plate method to measure the minimum inhibitory concentration (MIC) of honey to avoid the limitations of agar diffusion, where large, nonpolar polyphenols (which will not diffuse efficiently) play an important role in bioactivity. This study demonstrated that there are several alternative (non-manuka) honey types, particularly fresh raw Arkansas wildflower honeys, that comparably inhibit the growth of the antibiotic-resistant bacterial species specifically implicated in wound infections. Concentrations of 10–30% honey inhibited the growth of the highly antibiotic-resistant organisms colloquially referred to as “superbugs”, which the WHO declared in 2017 to be in critical need of new antibiotics. There was no statistical difference between manuka honey and fresh summer Arkansas wildflower honey in overall bacterial inhibition. These results could transform wound care in the United States, where manuka honey can be expensive and difficult to obtain and where antibiotic resistance remains a troubling concern for wound treatment.

## 1. Introduction

The Centers for Disease Control (CDC) estimate that approximately 2 million antibiotic-resistant healthcare-acquired infections (HAIs) are contracted annually in the United States, costing the country over $20 billion per year [1]. Antibiotic-resistant infections can be deadly, with approximately one tenth of these infections associated with mortality [1]. Surgical site infections (SSIs) and other wound infections together make up the largest percentage of the annual total of HAIs, about 25% [1]. These infections are caused by agents that have some of the highest rates of antibiotic resistance, such as *Acinetobacter baumannii*, which causes antibiotic-resistant infections in an alarming 33% of its cases [2]. Newborns, elders, and immunocompromised patients are especially vulnerable to these superbug infections.

With no new classes of antibiotics to effectively address antimicrobial resistance, antibiotic development research is widely pursued; however, microorganisms inevitably evolve resistance mechanisms to these new antibiotics. Hence, with the growing problem of antibiotic resistance, the need for alternative antimicrobial therapies has become critical. The focus of this project was to bring additional attention to the potential of natural bee honey as a promising alternative/supplement for the treatment of antibiotic-resistant wound infections and SSIs.

While honey is primarily a food product, it has also been historically used as an antibacterial wound salve, a cough medicine, a gingivitis treatment, and even a dietary supplement [3]. As a dietary supplement, honey contains a number of beneficial probiotics, prebiotics, minerals, and vitamins [4]. However, the botanical source of the nectar that the honey originates from determines its exact composition, so honey composition varies widely across different geographical and botanical origins [5,6].

Honey is essentially a digestion product of nectar by the western honeybee, *Apis mellifera*. Honeybees ingest the nectar of flowers, and then the sugars and phenols from the nectar are mixed with enzymes and peptides in a special bee storage organ called the crop. The mixture is regurgitated into honeycombs, where the bees concentrate it into honey by beating their wings to facilitate evaporation, leaving a viscous sugary solution containing no more than 15–20% water. The bees then seal the honey into the combs by capping each compartment with beeswax, and it can be stored there indefinitely as a food source for the bees during times of scarcity [5]. As long as the honey remains sealed away from moisture, it will not spoil, and microbes will not grow. This particular property of honey is the focus of recent scientific attention.

Honey contains an excess of glucose, sucrose, and fructose, and a bee-originating enzyme called glucose oxidase [6]. Glucose oxidase forms hydrogen peroxide as it oxidizes the glucose in honey under specific environmental conditions [6,7]. Hydrogen peroxide is a natural antimicrobial agent that is commonly used to disinfect wounds, and it is thought to be the primary mechanism by which honey inhibits the growth of bacteria [7,8]. However, the concentrations typically used for wound treatment are relatively cytotoxic compared to the concentrations found in honey. The concentration of peroxide found in honey is much lower and more cytocompatible, yet still as effective at bacterial inhibition [4].

While honey remains sealed, glucose oxidase is inactive in the acidic environment of undiluted honey (pH 3.2–4.5) [6,9]. The reaction between glucose and glucose oxidase occurs only when the pH is elevated and in the presence of sodium ions. Since the pH and Na^+^ content of skin is relatively high, an external wound provides the appropriate environment for this reaction to occur [10]. As honey becomes diluted by wound exudate, the aqueous exudate triggers the reaction to take place, and the result of honey in an open wound is the slow release of hydrogen peroxide over time as the wound exudes [10]. This is more effective at bacterial inhibition than a pure solution of hydrogen peroxide, which is very quickly degraded by biological enzymes and thus has a limited, short-term antiseptic effect on open wounds [11].

Recent studies have found that hydrogen peroxide alone is not fully responsible for the bioactivity of honey [11]. Some honeys retain antibacterial efficacy after the removal of the peroxide [4]. The predominant theory is that the polyphenols, peptides, and ions present in honey augment the effects of peroxide and sometimes work independently of it [7]. Several different honey-derived antimicrobial peptides and phenols have been identified with varied mechanisms of bacterial growth inhibition. For example, honey has the ability to inhibit biofilm formation in species that use it as a mechanism of resistance (*Staphylococcus aureus*, *Escherichia coli*, and *Proteus mirabilis*) [12,13,14]. Concentrations of honey as little as 0.5% have also has been implicated in the inhibition of quorum sensing, which is an important bacterial communication process used to coordinate virulence and resistance [15,16]. Additionally, honey inhibits the expression of important virulence genes, such as fibronectin binding proteins in *Streptococcus pyogenes* and curli genes (csgBAC), quorum sensing genes (AI-2 importer and indole biosynthesis), and virulence genes (LEE genes) in *E. coli* [14].

Manuka honey was the first FDA-approved honey type for wound treatment and is the most common source of medical-grade honey. Manuka honey has clinically demonstrated its ability improve the rate of wound healing compared to conventional wound dressings [17]. The results were considered clinically significant, although the study self-admittedly lacked the robustness needed for statistical significance [17]. 

Other honey types are not typically used for medical purposes and are less commonly included in antibacterial research. One large review comparing the minimum inhibitory concentrations (MICs) of manuka with those of other honeys has shown that there is generally a <5% difference between the antibacterial activity of manuka honey and other types of honey, which Hussain suggests can certainly be considered a clinically insignificant difference [4]. Lusby et al. reported that three other obscure honey types (lavender, red stringybark, and Paterson’s curse) have antibacterial activity equivalent to manuka for several bacterial species [18].

Manuka honey, derived from the Manuka bush (*Leptospermum scoparium*), is thought to be special because of its unique antibacterial compound, methylglyoxal (MGO) [10]. Since manuka is relatively low in hydrogen peroxide content, MGO has been considered the main driving force behind manuka’s antimicrobial properties; however, it has been shown that MGO concentration is not a reliable indicator of a honey’s antibacterial activity because it requires synergism with other components of honey to be effective as an antimicrobial agent [9,19]. Other non-manuka honeys may be more affordable, accessible, and equally effective in the treatment of wound infections, especially when combined with other therapies [20].

Since previous studies have shown that honey’s antimicrobial efficacy is multi-mechanistic in nature, we hypothesized that any honey type would inhibit the growth of even the most hypermutable strains of wound-infecting, highly antibiotic-resistant bacteria. We also hypothesized that wildflower honeys would be more effective than monoflorals because of a greater diversity of polyphenols (similar to how a polypharmacological approach is more efficacious than single drug therapy alone for highly antibiotic-resistant infections). Thus, manuka honey, which is relatively expensive and difficult to obtain in large quantities, could be joined by other more accessible honeys on the list of FDA-approved wound treatments.

Using MIC assays via honey plates, which expose organisms to known honey concentrations and do not require chemical diffusion, we analyzed organisms with antibiotic resistance that are on the World Health Organization’s (WHO) list of organisms that need an antibiotic alternative [21]. The aim of this study was to identify alternative (non-manuka) honey types that can be specifically used against antibiotic resistance bacteria in wound infections. We used over fifty-five honey samples, including the monoflorals of manuka, clover, acacia, and orange blossom. We also used a variety of wildflower honeys, including freshly produced Arkansas honey. Our results indicate that fresh Arkansas honey from a summer harvest is statistically equivalent to manuka honey. In our experiments, pH had little effect on MIC, honey types with increased osmolarity had higher MICs, and darker colored honeys had lower MICs. Finally, our results indicate the choice of honey for a prospective wound treatment is dependent upon the infectious organism. Comparing manuka honey to summer and spring harvest Arkansas wildflower honeys, we found *Pseudomonas aeruginosa* to be equally sensitive across all three honey types. *Klebsiella pneumoniae*, *E. coli*, and *Enterobacter aerogenes* were inhibited with either manuka or summer harvest Arkansas honeys, while *A. baumannii* and *S. aureus* in the form of BORSA and VISA were most effectively inhibited with manuka. Furthermore, *P. aeruginosa* and *A. baumannii* were more sensitive to honey exposure compared to the other species tested, despite the higher antibiotic resistance rates of these organism [2]. It is apparent that honey’s antibacterial efficacy is not only dependent upon floral source but also the microbial species being treated, which will ultimately be important considerations for potential future medical applications.

## 2. Results and Discussion

### 2.1. Antibacterial Efficacy Assays

**Figure 1:**
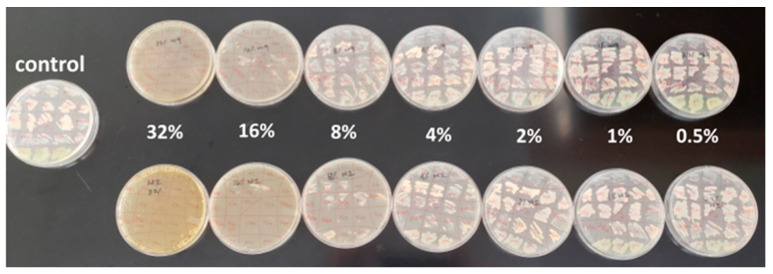
Example of a honey plate MIC assay. Each organism is patched onto the plates in triplicate. The MIC is defined as the lowest concentration of honey at which an organism does not grow. The three values obtained in one experiment are averaged in order to control for differences in inoculation volume that may occur in this dry patch method. Plates are Mueller–Hinton media with honey added to equal 0.5–32% honey, while the control is Mueller–Hinton media with 0% honey.

There are a number of different ways to determine the minimum inhibitory concentration (MIC) of a substance. It is very common to use a disc- or well-diffusion assay for typical polar, hydrophilic compounds; however, this methodology limits the movement of large, plant-derived nonpolar compounds and phenols that are thought to play an important role in honey bioactivity. Agar diffusion is a qualitative, relative method of measurement, where the result depends largely on the ability of the substance to diffuse well. A zone of inhibition that seems small could give a false impression that the honey is not effective. Using the honey plate method, we exposed the bacteria in this study to a uniform, known concentration of each honey and were able to assay a large number of bacterial replicates at a time, which conserved time and resources while maintaining experimental quality (Figure 1).

Using the honey plate assay, we hypothesized that wildflower honey would be more effective as an antibacterial agent because it presumably contains a wider variety of antibacterial botanical compounds. Since manuka honey is already a well-known FDA-approved wound treatment, we included manuka honey in this study as a comparative standard.

**Figure 2:**
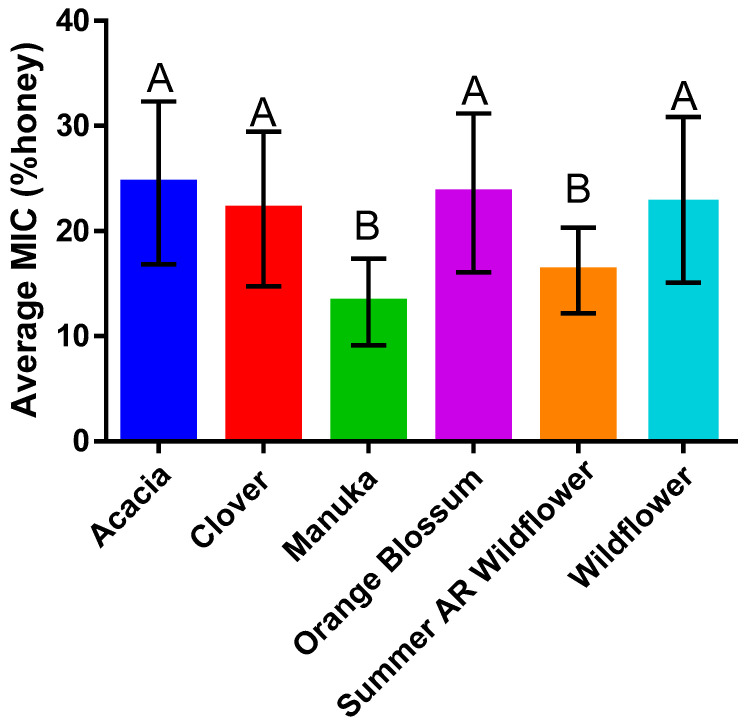
Comparison of honey type (*n* = 46) and average MIC for all bacteria species (*n* = 7). There was a significant effect of honey type on average MIC (Kruskal–Wallis, X^2^ = 102.8, df = 5, *p* < 0.0001) (mean ± SD). Overall, manuka (*n* = 12) and summer Arkansas wildflower (*n* = 9) had the highest efficacy and demonstrated statistical equivalence in comparison to the poorest performing honeys, acacia (*n* = 5), clover (*n* = 6), orange blossom (*n* = 7), and wildflower (*n* = 7), which were not statistically different from one another. These results highlight how floral source differences impact microbial inhibition and suggest that manuka and summer Arkansas wildflower are comparable in antimicrobial efficacy despite their differences in floral source (Dunn’s, Supplemental Table S1). Columns with equivalent letters (e.g. A and A) are statistically similar while columns with different letters (e.g A and B) are statistically different.

Overall, honey floral source impacted antibacterial efficacy (Kruskal–Wallis, *p* < 0.0001) (Figure 2). Monofloral honeys, in this study, were generally less effective inhibitors of bacteria, except for manuka. Packaged wildflower honey was generally more similar to the monofloral honeys in efficacy, ranking as less effective than freshly harvested summer-season Arkansas wildflower honey (Figure 2).

Manuka consistently inhibited bacteria at a marginally lower concentration than all other honey types tested; however, the clinical and statistical significance of that observation remains in question. For every organism tested, manuka honey and Arkansas summer wildflower honey were considered statistically the same. The difference in MIC between these two honey types never exceeded 8.1% across all organisms tested. This implies that manuka honey is not the only honey type that could potentially present a solution to the problem of antibiotic resistance.

These results somewhat support our original hypothesis that wildflower honey would be more effective than other monoflorals, since summer Arkansas wildflower honeys were statistically equivalent to manuka, although there is more here to investigate. The manuka honeys obtained for this study were packaged and sealed similarly to the “other” wildflower honeys, and only the fresh, raw local honey was equal to manuka in efficacy. Perhaps manuka honey has a more stable shelf life than wildflower honeys, or perhaps the packaging process of manuka honey differs from honeys packaged for typical market shelves. It will be important to determine in future studies how the packaging process or shelf-life impacts the chemical make-up of different honey types.

### 2.2. The Role of pH, Osmolarity, and Color in Honey Antibacterial Efficacy

**Figure 3:**
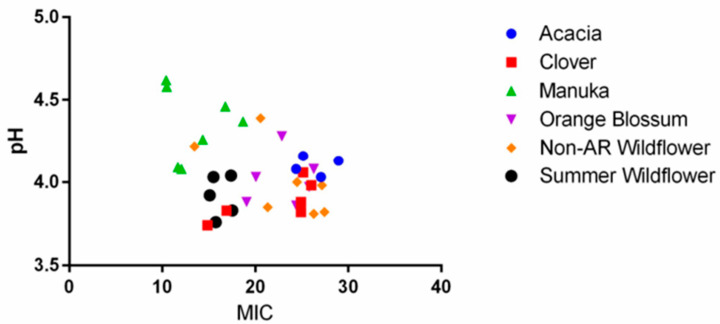
Relationship between a honey’s pH and average MIC against all species tested. There is a weak correlation between MIC and pH (Spearman’s R = −0.2031, *p* = 0.166, *n* = 48), such that as pH increases, honey becomes more effective overall (smaller MIC value).

The antibacterial ability of honey has been known for thousands of years, but how it works has remained a scientific conundrum. We measured pH and color (using spectrophotometric analysis) of our honeys to identify the nature of the relationship between these factors and MIC. We hypothesized, in agreement with other published theories on honey’s antibacterial nature, that lower pH is an important contributor to honey’s antibacterial effect [9]. When we examined the correlation between pH and MICs obtained via our honey plate assay, we found there to be only a very weak correlation (Spearman’s R = −0.2031), trending in the opposite direction of what was expected (Figure 3).

As pH increased in our honeys, they became slightly more effective overall. Honey has an average pH range of 3.2–4.5 but can be as high as pH 6.1 according to the National Honey Board [22]. The pH of our honeys had a narrow range of 3.7–4.7, which makes it less likely that we can detect a correlation between pH and MIC. This correlation is not typically investigated, presumably because there has always been a reasonable assumption that greater acidity would result in greater cell death. In this study, the honeys with the highest pH were mostly manuka honeys, and their pH values were consistent with those reported by Roshan et al. [23]. The trend we found suggests that the compounds in honey that may be responsible for increased bioactivity may also decrease honey’s acidity. Since the oxidation of glucose into hydrogen peroxide occurs at a less acidic pH, this observation is consistent with the many publications that attribute hydrogen peroxide activity to honey’s antibacterial efficacy [11].

**Figure 4:**
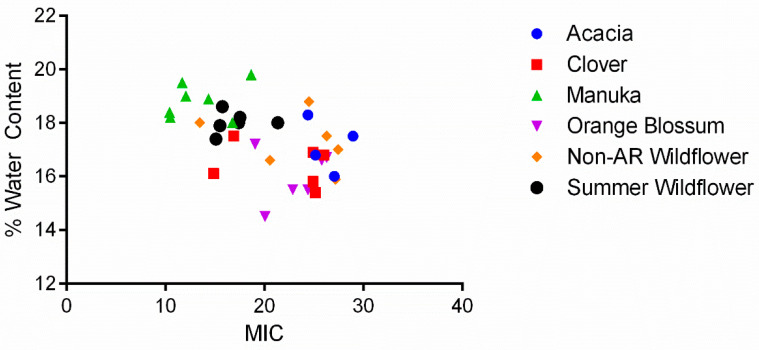
Relationship between a honey’s water content and average MIC against all species tested. There is a significant inverse trend, indicating that a higher water content corresponds to a higher efficacy (lower MIC) (Pearson’s R = −0.543, *p* < 0.0008, *n* = 35).

Like the assumptions about honey’s acidity, it has been theorized in countless honey-related research introductions that honey’s high osmolarity is one of the primary contributors to its antibacterial effect [9]. We hypothesized the same, but instead found that the most effective honeys tended to have a lower osmolarity (Pearson’s R = −0.543, *p* < 0.0008, *n* = 35) (Figure 4). Molan and Rhodes explained that glucose oxidation occurs at a higher rate in the presence of water molecules (increasing the production of hydrogen peroxide) [10]. This correlates with our findings that lower osmolarity increased antimicrobial activity. The range of percent water content of our honeys was again too narrow to determine exactly when the effect is lost; however, our results suggest that glucose oxidation may play a more important role in honey’s bioactivity than osmotic pressure.

**Figure 5:**
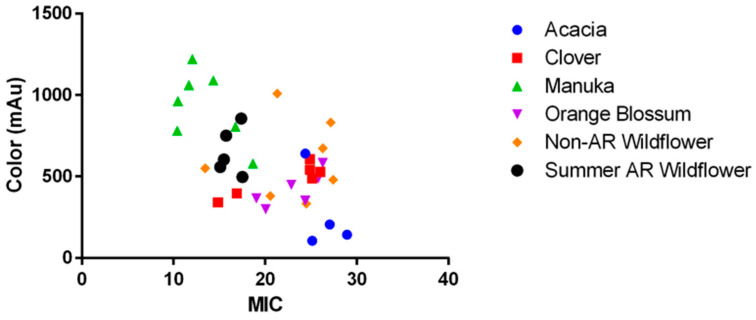
Relationship between a honey’s color (as measured by spectrophotometric analysis) and average MIC against all species tested. There is a moderate correlation (Pearson’s R = −0.581, *p* < 0.0001, *n* = 38) where darker colored honeys inhibit bacteria at a lower MIC.

Another common assumption about honey (and better studied, for that matter), is that darker-colored honeys contain more bioactive components such as phenols, amino acids, and enzymes that would inhibit bacterial growth [24]. Our results show darker-colored honeys were moderately associated with lower MICs (Pearson’s R = −0.581, *p* < 0.0001, *n* = 38) (Figure 5), which is in line with current literature trends. This moderate association between color and MIC was the strongest compared to other correlations tested in this study and indicates that these bioactive compounds play a significant role in honey’s antibacterial efficacy. Some of these compounds have been identified, isolated, and shown to have antibacterial activity alone [20,25,26,27], but none produce the level of bioactivity of honey itself, suggesting that there are other potentiating effects to consider.

### 2.3. Harvest Timing Determines Antimicrobial Activity

**Figure 6:**
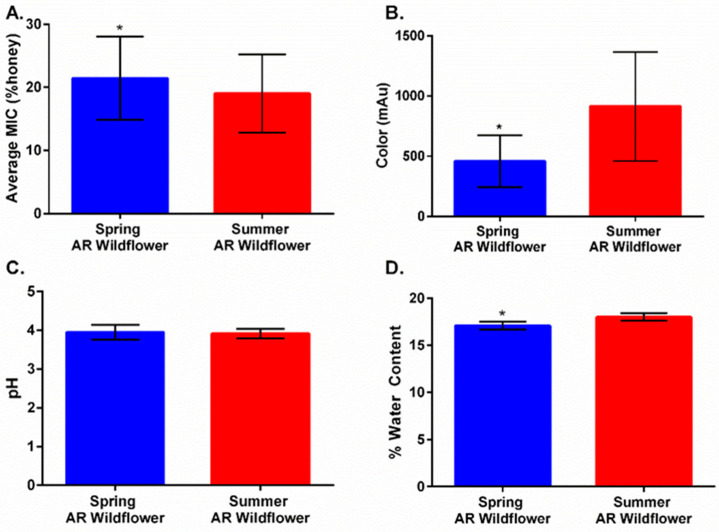
Comparison of average MIC, color, pH, and water content between spring and summer Arkansas wildflower (mean ± SD). (**A**) Average MIC across all bacterial species (*n* = 7) varied significantly between spring (*n* = 91) and summer honeys (*n* = 56). Spring honeys had a significantly higher average MIC when compared to summer honeys (Mann–Whitney, *p* = 0.0225, U = 1987). (**B**) Comparisons of color yielded a significant difference between the two Arkansas wildflower honey harvests (unpaired *t*-test, *p* = 0.0167). Spring honeys (*n* = 9) had a significantly lighter color compared to summer honeys (*n* = 8). (**C**) No significant difference between spring honeys (*n* = 13) and summer honeys (*n* = 5) was observed when pH was evaluated (unpaired *t*-test, *p* = 0.7212). (**D**) When comparing water content, there was a significant difference between spring and summer (unpaired *t*-test, *p* = 0.0302). Spring honeys (*n* = 2) were significantly lower in water content compared to summer honeys (*n* = 6). * indicates significant difference.

In order to explore similarities between manuka and local wildflower honeys, we gathered additional local honey samples from donors across the state of Arkansas. When comparing MICs of the original Arkansas honeys alongside the new honey samples, these new additions were unexpectedly not as effective (Figure 6a). Analysis of harvest date indicated most of our original honeys were from a summer harvest, while the newest honeys were from a spring harvest. Subsequently, we compared MIC, color, pH, and water content of the honey samples based on harvest (Figure 6). Samples harvested in summer had a significantly lower average MIC, were darker in color, and had a higher water content compared to samples harvested in spring (Figure 6a,b,d), while pH remained relatively the same (Figure 6c). These differences can be attributed to seasonal floral availability, which can vary greatly between seasons and impact bioactive components of honey. During spring, floral availability greatly increases, as numerous wildflower species begin to bloom, and it tends to decline as summer transitions into the fall season. A few noteworthy Arkansas wildflower species include Bird’s Foot Violet (March–May), Blue Star (April–June), Blackeyed Susan (May–October), and Tickseed (August–November) [28,29]. These findings support how honey’s floral origins play a crucial role in antimicrobial activity, which is greatly dependent upon floral source, making harvest time an important factor in overall composition and performance.

### 2.4. Honey Type Efficacy Is Species-Dependent

**Figure 7:**
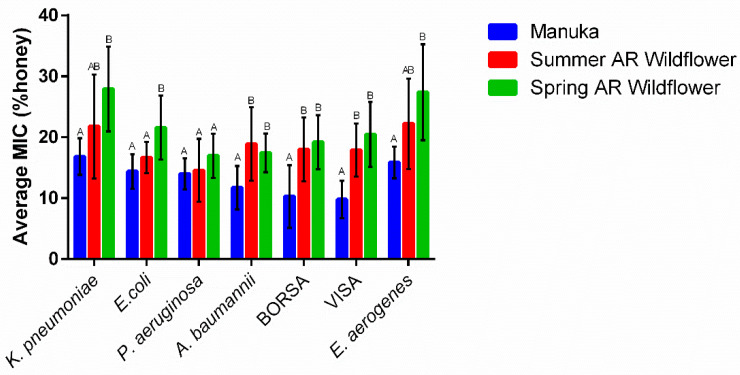
Comparison of the average MICs for each bacteria species tested (mean ± SD). The MICs of manuka and spring Arkansas honey were significantly different for *K. pneumoniae*, *E. coli*, *A. baumannii*, BORSA, VISA, and *E. aerogenes* (one-way ANOVA, Tukey; *p* = 0.0005, *p* = 0.0002, *p* = 0.0047, *p* = 0.0002, *p* < 0.0001, *p* = 0.0003, respectively). Manuka is also significantly more effective than summer Arkansas honey for *A. baumannii*, BORSA, and VISA (one-way ANOVA, Tukey; *p* = 0.002, *p* = 0.0043, *p* = 0.001 respectively), which are generally the most sensitive organisms. Summer Arkansas honey is only significantly different from spring Arkansas honey for *E. coli* (one-way ANOVA, Tukey *p* = 0.0238), and there is no significant difference across honey types for *P. aeruginosa* (one-way ANOVA, *p* = 0.1450). Additional *p*-values are in Supplemental Table S2. Columns with equivalent letters (e.g. A and A) are statistically similar while columns with different letters (e.g A and B) are statistically different.

The focus of this study was to examine alternative honey types as antibacterial treatment potential for microbes most frequently associated with antibiotic-resistant wound infection. In the process, we showed that alternative honeys do exist, but floral source, honey osmolarity, and color determine antimicrobial effectiveness. Using our most antimicrobial honeys, manuka, spring Arkansas wildflower, and summer Arkansas wildflower, we compared antimicrobial activity across our microbial species (Figure 7). We analyzed organisms that are known causes of nosocomial antibiotic-resistant infections and are classified as organisms of concern by WHO [22,30]. The results of our study show that the efficacy of varying honey types is also dependent on species, suggesting that there are different mechanisms of antibacterial action in play for each species of microbe (Figure 7). For some microbes, such as *P. aeruginosa,* the honey type does not matter—all of the honeys tested yielded statistically similar MICs. Carbapenem-resistant *E. aerogenes* and *K. pneumoniae* were equally susceptible to manuka and summer Arkansas wildflower honey. *E. coli* was equally susceptible to manuka and summer Arkansas wildflower honey, but spring Arkansas wildflower honey efficacy was significantly different.

In terms of organism sensitivity, *S. aureus*, which happens to be the most commonly studied species in honey antibacterial research, was most sensitive to manuka of all the species tested (BORSA MIC = 10.3%, *n* = 12 and VISA MIC = 9.8%, *n* = 12). *P. aeruginosa*, a known hypermutable species, was most sensitive to honey overall (MIC = 15.3%, *n* = 34), while *K. pneumonia* (MIC = 22.2%, *n* = 32) and *E. aerogenes* (MIC = 22%, *n* = 31) were our most resistant organisms. Interestingly, these two species occur at similar SSI rates and also demonstrate similar carbapenem resistance rates (about 3% of isolates tested) [2]. In the 2016 Weiner et al. report, carbapenem-resistant Enterobacteriaceae (CREs) have lower carbapenem resistance rates than *Pseudomonas* and *Acinetobacter*, which resist carbapenems at a rate of 7.7% and a remarkable 33.3% (respectively) of isolates tested [2]. However, in our study, the CREs (*E. coli*, *E. aerogenes,* and particularly *K. pneumoniae*) were noticeably more resistant to honey treatment than *P. aeruginosa* and *A. baumannii.*

*A. baumannii* responded strongly to honey exposure; this species was consistently inhibited by all honeys at 11–19% concentrations. *A. baumannii* is especially problematic as a healthcare-associated infectious agent. It is not the most common SSI agent, but it has a very high rate of resistance against all known antibiotics [2]. *A. baumannii* is not ubiquitous nor a part of normal flora; it only exists as an HAI [31]. In light of all this, it is especially noteworthy that honey seems to work well against *A. baumannii* (Overall MIC = 15.7%, *n* = 33).

BORSA and VISA were included in this study because *S. aureus* remains the most common causative agent of SSI and is Priority 2 (high) on the 2017 WHO Priority Pathogens List for R&D of New Antibiotics [2,21]. Predictably, the results for both BORSA and VISA were very similar to each other. They followed the same pattern of floral source dependence in that they were best inhibited by manuka and summer Arkansas wildflower honeys. Overall, compared to the other species tested, *S. aureus* (MIC = 15.7%, *n* = 60) tended to be more susceptible to honey than the CREs (MIC = 22%, *n* = 64).

Manuka honey and Arkansas wildflower honey inhibited the growth of all the multidrug-resistant bacteria species in this study even when highly diluted. In wound care, it is important to consider a dressing that can maintain efficacy as the wound exudate naturally dilutes the topical treatment over time. The MICs in this study are for complete inhibition of growth and do not take into account that at even lower concentrations, honey is still inhibiting a large degree of bacterial growth, while also interfering with virulence factors and facilitating wound healing through mechanisms related to re-epithelialization, immune stimulation, and so on [3]. Given this, a honey dressing would continue to provide a significant advantage to a person with an antibiotic-resistant wound infection even if the wound is exuding heavily.

Wound infections are second only to hospital-acquired pneumonia in healthcare infection prevalence [1]. HAIs continue to be a serious problem, which has been exacerbated by the increasing occurrence of antibiotic resistance. Weakened surgical patients, burn patients, diabetics with open ulcers, and elderly immobile patients with bedsores are especially vulnerable to these resistant wound infections in healthcare facilities. The overuse of antibiotics combined with the weakened immunity of this demographic contributes to a high rate of mortality for those who acquire wound infections [32,33].

Our study shows that different honey types fully inhibit clinical, antibiotic resistant isolates at relatively low concentrations—15–25%, a concentration low enough to theoretically tolerate even a heavily exuding wound. Furthermore, complete inhibition is not necessary for the successful life-saving treatment of a resistant infection. Honey weakens bacteria while strengthening the immune system [34,35], and becomes more active as it is diluted by exudate [4]. Honey reverses resistance even at sub-inhibitory concentrations [36], improves the efficacy of existing drugs when used in combination therapy [27], and seems to prevent bacteria from developing new resistance mechanisms [37]. These properties of honey can give patients a life-saving advantage in the face of a life-threatening disease, even when it has been diluted beyond 80%.

## 3. Conclusions

Our research shows that less expensive, more accessible honey types (non-manuka) have the potential to contribute the same advantages to wound treatment that manuka has shown in recent research. Fresh summer Arkansas wildflower honey in particular was consistently comparable to manuka honey in our study. Freshness likely plays an important role in this observation, but we hope further chemical analysis of the honeys will provide more information. It seems likely that some of the compounds contributing to antibacterial activity in diluted honeys may be degraded in processed, packaged wildflower honeys, while the multifloral honeys obtained raw from local apiaries maintained manuka-equivalent activity. Additionally, the findings of this study suggest floral source and bacterial species will respond differently to each other (and just like any laboratory-developed drug, one treatment may be more appropriate for some infections over others).

Ultimately, the goal of this study was to make progress in the field of antibiotic resistance research by investigating an alternative to traditional failing medications. Honey could save lives and billions of US dollars each year if it were more widely available, backed by well-controlled clinical trials, and more advertised in the medical community. Past research has focused almost entirely on the benefits of manuka honey, which is relatively expensive and not widely available in the United States. More research is needed that compares other honey types to manuka using appropriate MIC assay methods against the bacterial species most in need of antibiotic alternatives. These studies could transform wound care in the United States, making life-saving options available where there was once very little hope.

## 4. Materials and Methods

### 4.1. Microorganisms

The organisms for this study were chosen based on their prevalence in nosocomial wound infections, their high rate of antibiotic resistance [30], and their presence on the list of organisms that have been declared by the WHO [21] to be in the most critical need of an antibiotic alternative. We obtained the following organisms from the CDC and stored them at −80 °C for the duration of the study:

Multi-drug-resistant *Pseudomonas aeruginosa;*

Carbapenem-resistant *Acinetobacter baumannii;*

Carbapenem-resistant *Klebsiella pneumonia;*

Carbapenem-resistant *Escherichia coli;*

Carbapenem-resistant *Enterobacter aerogenes;*

Oxacillin-resistant *Staphylococcus aureus* (BORSA);

Vancomycin-intermediate *Staphylococcus aureus* (VISA).

Stock cultures of these organisms were used for MIC assays and maintained on sheep’s blood agar (SBA) for no longer than 1 month at 4 °C before being subcultured. Cultures were not subcultured more than three times. As needed, fresh cultures were grown from freezer stock by scratching the frozen broth culture with a sterile loop and streaking onto SBA. The carbapenem-resistant Enterobacteriaceae (CRE) we selected for this study were chosen based on their current high prevalence rate in healthcare-acquired infections; *Klebsiella, E. coli*, and *Enterobacter* rank in the top five causative agents of SSIs, with 1–5% of the isolates tested from 2011–2014 demonstrating resistance to all known antibiotics [2,38]. In 2017, these carbapenem-resistant species were placed on the WHO Priority Pathogens List for R&D of New Antibiotics, Priority 1 (critical), along with carbapenem-resistant *A. baumannii* and *P. aeruginosa* [21].

### 4.2. Honey Sample Acquisition and Processing

The honeys used in this study were either donated or purchased from both local and non-local sources. When necessary, crystallized honey samples were gently warmed to approximately 50 °C to liquefy, so that they could be aliquoted for measuring osmolarity, color, and pH. The honey samples were kept at ambient temperature under interior lighting in sealed containers.

### 4.3. Honey pH

For each honey sample, 2.664 +/−0.005 g honey was weighed into a 50 mL beaker. We dispensed 20 mL CO_2_-free deionized water into the beaker and added a stir magnet. The beaker was covered with a watch glass, transferred to a magnetic stir plate, and stirred on high for approximately 5 min. The pH of the solution was measured using a Sartorius pHBasic (Sartorius, Göttingen, Germany) pH meter according to the methods described by the International Honey Commission [39].

### 4.4. Osmolarity Determinations

The osmolarity of each sample was represented by the percent water content. We determined the water percent for each honey sample using a Brix refractometer (V-Resourcing, Amazon), following the International Honey Commission methods published in 2002 [39]. In short, the refractive index (RI) for each honey sample was measured twice and then averaged. RI corresponds to a water content (%) value as published by the International Honey Commission [39].

### 4.5. Honey Color

Color intensity of each honey sample is defined by Beretta et al., as the difference in absorbance of a 50% honey solution at 450 nm and 720 nm [40]. Each sample was diluted 50% (*v*/*v*) with warm (50 °C) distilled water, vortexed, and pipetted into a microplate. Absorbance was measured at both 450 nm and 720 nm using a Synergy H1 microplate reader (Aglient, Santa Clara, CA, USA), with 450 nm measuring the presence of honey pigments, carotenoids, and phenolics. The difference in absorbances was represented as mAU.

### 4.6. Honey Type

The honey type for each sample was determined by packaging information or else declared by the provider. The honeys were typed according to their botanical source by genus. Samples for which floral source could not be determined were not used in this study. Arkansas wildflower honey was categorized separately from store-bought wildflower honey because of noticeable differences in MIC and ^1^H NMR spectra that were observed in the preliminary data [41].

### 4.7. Minimum Inhibitory Concentration (MIC) Assays

In order to overcome the limitations of agar diffusion assays, aliquots of each honey sample were uniformly dissolved into cooled (approximately 55 °C), sterile, molten Mueller–Hinton agar and then poured into a sterile petri dish, creating “honey plates”. These plates were made at the following concentrations of honey (*v*/*v*): 32%, 16%, 8%, 4%, and 2%. We patched the antibiotic-resistant bacterial species obtained from the CDC from a sheep blood agar culture directly onto small sections of each honey plate in triplicate. The lowest honey plate concentration at which each species did not grow was considered the minimum inhibitory concentration (MIC) for that species (recorded as the average of the three technical replications). This experiment was performed in triplicate for each honey sample on different days. The negative control consisted of an un-inoculated patch on each plate, while the positive control was a standard Mueller–Hinton agar plate inoculated as per the protocol. We tested fifty-nine total honey samples representing six floral sources (local raw AR wildflower, packaged/processed store-bought wildflower, manuka, orange blossom, clover, and acacia).

### 4.8. Statistics

All statistical analyses were done in GraphPad Prism version 6.07 for Windows (GraphPad Software, La Jolla California USA, www.graphpad.com, accessed on 15, March 2022). In all cases, an alpha of 0.05 was deemed significant.

We used Kruskal–Wallis with Dunn’s post hoc to compare honey types to MIC averaged for all six microorganisms. Each honey sample was tested three times; the following are the number of samples of each honey we tested: acacia *n* = 5, clover *n* = 6, manuka *n* = 12, orange blossom *n* = 7, summer Arkansas wildflower *n* = 8, non-Arkansas wildflower *n* = 14. To determine correlations between pH, osmolarity, or color with MICs, we used Spearman’s correlation for nonparametric data and Pearson’s correlation for parametric data: pH *n* = 48, osmolarity *n* = 35, color *n* = 38. To compare summer Arkansas wildflower honey and spring Arkansas wildflower honey MICs (spring *n* = 91, summer *n* = 56), pH (spring *n* = 13, summer *n* = 5), osmolarity (spring *n* = 2, summer *n* = 6), and color (spring *n* = 9, summer *n* = 8), unpaired *t*-tests were used for parametric data and Mann–Whitney was used for nonparametric data. One-way ANOVA with Tukey’s post hoc was used to compare manuka, summer Arkansas wildflower honey, and spring Arkansas wildflower honey to six different microorganisms.

## Data Availability

The data presented in this study are available on request from the corresponding author.

## Supplementary Materials

The following supporting information can be downloaded at: https://www.mdpi.com/article/10.3390/antibiotics11081132/s1, Table S1: Dunn’s post hoc *p*-values for comparison of honey type and average MIC for all bacteria species; Table S2: One-way ANOVA, Tukey’s post hoc *p*-values for comparison of Manuka, summer Arkansas wildflower honey, and spring Arkansas wildflower honey to six different microorganisms.
