# Supplementary material for: Both Manuka and Non-Manuka Honey Types Inhibit Antibiotic Resistant Wound-Infecting Bacteria
Source: Antibiotics (Basel). 2022 Aug 20;11(8):1132. doi: 10.3390/antibiotics11081132 (PMC9405051; doi:10.3390/antibiotics11081132)
Supplement: Supplementary file 1 [file antibiotics-11-01132-s001.zip › antibiotics-1860507-supplementary.pdf]

antibiotics

MDPI

Supplemental Table S1. Dunn's post hoc p-values for comparison of honey type and average MIC for all bacteria species.

Comparison	p-value
Acacia vs Clover	>0.9999
Acacia vs Manuka	<0.0001*
Acacia vs Orange Blossom	0.9999
Acacia vs Summer Arkansas Wildflower	0.0002*
Acacia vs Non Arkansas Wildflower	>0.9999
Clover vs Manuka	<0.0001*
Clover vs Orange Blossom	>0.9999
Clover vs Summer Arkansas Wildflower	0.0388*
Clover vs Non Arkansas Wildflower	>0.9999
Manuka vs Orange Blossom	<0.0001*
Manuka vs Summer Arkansas Wildflower	0.3850
Manuka vs Non Arkansas Wildflower	<0.0001*
Orange Blossom vs Summer Arkansas Wildflower	0.0011*
Orange Blossom vs Non Arkansas Wildflower	>0.9999
Summer Arkansas Wildflower vs Non Arkansas Wildflower	0.0056*

* indicates significant differences

Supplemental Table S2. : One-way ANOVA, Tukey's post hoc p-values for comparison of Manuka, summer Arkansas wildflower honey, and spring Arkansas wildflower honey to six different micro-organisms.

Organism	Comparison	p-value
K. pneumoniae	Manuka vs Summer Arkansas Wildflower	0.2104
	Manuka vs Spring Arkansas Wildflower	0.0005*
	Summer vs Spring Arkansas Wildflower	0.0975
E. coli	Manuka vs Summer Arkansas Wildflower	0.4304
	Manuka vs Spring Arkansas Wildflower	0.0002*
	Summer vs Spring Arkansas Wildflower	0.0238*
P. aeruginosa	Manuka vs Summer Arkansas Wildflower	0.9418
	Manuka vs Spring Arkansas Wildflower	0.1453
	Summer vs Spring Arkansas Wildflower	0.3509
A. baumannii	Manuka vs Summer Arkansas Wildflower	0.0020*
	Manuka vs Spring Arkansas Wildflower	0.0047*
	Summer vs Spring Arkansas Wildflower	0.7236
BORSA	Manuka vs Summer Arkansas Wildflower	0.0043*
	Manuka vs Spring Arkansas Wildflower	0.0002*
	Summer vs Spring Arkansas Wildflower	0.8496
VISA	Manuka vs Summer Arkansas Wildflower	0.0010*
	Manuka vs Spring Arkansas Wildflower	<0.0001*
	Summer vs Spring Arkansas Wildflower	0.4066
E. aerogenes	Manuka vs Summer Arkansas Wildflower	0.0846
	Manuka vs Spring Arkansas Wildflower	0.0003*
	Summer vs Spring Arkansas Wildflower	0.1825

* indicates statistical differences.
